# The Tinnitus Research Initiative (TRI) database: A new approach for delineation of tinnitus subtypes and generation of predictors for treatment outcome

**DOI:** 10.1186/1472-6947-10-42

**Published:** 2010-08-03

**Authors:** Michael Landgrebe, Florian Zeman, Michael Koller, Yvonne Eberl, Markus Mohr, Jean Reiter, Susanne Staudinger, Goeran Hajak, Berthold Langguth

**Affiliations:** 1 Department of Psychiatry, Psychosomatics and Psychotherapy, University of Regensburg, Universitaetsstraße 84, 93053 Regensburg, Germany; 2 Center for Clinical Studies, University Hospital Regensburg, Franz-Josef-Strauss-Allee 11, 93053 Regensburg, Germany; 3 ManaThea GmbH, Josef-Engert-Straße 11, 93053 Regensburg, Germany

## Abstract

Tinnitus, the phantom perception of sound, is a frequent disorder that causes significant morbidity and treatment is elusive. A large variety of different treatment options have been proposed and from most of them some patients benefit. However, a particular treatment that helps one patient may fail for others. This suggests that there are different forms of tinnitus which differ in their pathophysiology and their response to specific treatments. Therefore, it is a major challenge for tinnitus treatment to identify the most promising therapy for a specific patient.

However, most published clinical treatment studies have enrolled only relatively small patient samples, making it difficult to identify predictors of treatment response for specific approaches. Furthermore, inter-study comparability is limited because of varying methods of tinnitus assessment and different outcome parameters. Performing clinical trials according to standardized methodology and pooling the data in a database should facilitate both clinical subtypisation of different forms of tinnitus, and identification of promising treatments for different types of tinnitus. This would be an important step towards the goal of individualized treatment of tinnitus.

For these reasons, an international database of tinnitus patients, who undergo specific treatments, and are assessed during the course of this treatment with standardized instruments (e.g., psychoacoustic measures, questionnaires) has been established. The primary objectives of this database are (1) collecting a standardized set of data on patient characteristics, treatments, and outcomes from tinnitus patients consulting specialized tinnitus clinics all over the world (at present 13 centers in 8 countries), (2) delineating different subtypes of tinnitus based on data that has been systematically collected and (3) identifying predictors for individual treatment response based on the clinical profile. Starting in 2008, the database currently contains data from more than 400 patients. It is expected that more centers will join the project and that the patient numbers will rapidly grow, so that this international database will further facilitate future research and contribute to the development of evidence based on individualized treatment.

## Background

Chronic tinnitus is a frequent disorder characterized by high morbidity and a significant reduction in the quality of a patient's life [1,2]. Currently, manifold treatment strategies are in use [3,4]. Some of these approaches have been investigated in controlled trials [5]. In many of the controlled treatment trials, average improvement for the whole sample was negligible, frequently failing to reach statistical significance. However in many of these studies there were subgroups of patients for whom specific treatment strategies were effective. This applies to clinical trials investigating a large variety of different treatment interventions, including brain stimulation techniques [6-11], pharmacotherapy [3], psychotherapy [12] or auditory stimulation [13,14]. So far, the best evidence available for improving the quality of life in tinnitus patients is cognitive behavioural therapy [12]. Nevertheless, the results of most of these studies are characterized by a high interindividual variability of treatment response. On the one hand, factors not directly related to tinnitus (e.g., psychosocial stress, expectation and anticipation of treatment success, etc.) may contribute to the variability of treatment results. Indicators for such influences are e.g. the considerable variability of tinnitus severity over time irrespective of treatment interventions or the high variability of response to placebo treatments. On the other hand, the high variability in treatment results may reflect the pathophysiologic heterogeneity of the symptom tinnitus. Tinnitus can be the consequence of many different etiological conditions; it further varies in its perceptual characteristics (e.g., pulsatile vs. non-pulsatile, intermittent vs. permanent, uni- vs. bilateral tinnitus, pure tone vs. broadband noise, pitch, loudness, etc.) and in its comorbidities (hearing loss, hyperacusis, vertigo, auditory processing disorders, affective disorders etc.). It has to be assumed that these different forms of tinnitus differ in their pathophysiology and also in their response to specific treatments. Therefore, a "one fits all"-approach is not feasible in tinnitus therapy. Furthermore, clinical trials with inhomogeneous patient populations may reveal false negative results, if positive results in a small subgroup are diluted by negative results in the rest of the sample [15]. An example is the treatment with the anticonvulsant carbamazepine. Whereas several trials in unselected samples were negative [16,17], a specific form of tinnitus that is caused by vascular compression of the auditory nerve and characterized by a "type writer"-like sound, responds to carbamazepine [18,19]. This example indicates that higher treatment success can be achieved with specific treatment regimes for specific subgroups. For such an individualized approach, determination of predictors of treatment response is needed but still widely lacking so far.

Since tinnitus is a frequent and chronic disease and affected patients undergo in many cases different treatment approaches, the socio-economic costs of the disease are substantial. In order to reduce these costs and to alleviate the burden of the disease, a better predictor-based stratification of patients to treatment approaches, which hold a good chance of positive response in the individual case, is urgently needed. In order to reduce the heterogeneity of tinnitus, different forms of tinnitus should be grouped in subtypes based on e.g. etiologic factors, clinical appearance or co-morbid medical conditions. This may increase the chances of detecting effective treatments in distinct tinnitus forms. However, frequently at the time of the design of a clinical trial, it is not yet known which subgroup of tinnitus patients may benefit best from treatment. Moreover, a prerequisite for such an approach is a large, well characterised tinnitus sample.

The first attempt at systematically collecting data from tinnitus patients is the web-based *Oregon Tinnitus Data Archive *[20]http://www.tinnitusarchive.org/. This free, accessible database contains statistical data summaries of current status and past history of tinnitus, results of specific tinnitus tests, audiologic status and demographic and general information in a sample of 1630 tinnitus patients who attended the tinnitus clinic of the Oregon Hearing Research Center between 1981 and 1994. This database allows a description of basic tinnitus characteristics in a large patient sample. This database was intended to contribute to (1) better classification of tinnitus, (2) planning etiologic studies, (3) epidemiologic research, (4) treatment evaluation and (5) studies of tinnitus mechanisms. This pioneering work has contributed largely to a better understanding of tinnitus [21], but it has several important limitations. First, the *Oregon Tinnitus Data Archive *only contains cross-sectional data. Thus, it cannot provide information about the interaction between specific tinnitus characteristics and outcome of specific treatments. The lack of longitudinal data makes it impossible to use the database in the search for predictors of treatment outcome for specific treatment interventions. A second limitation of the database is the highly selected patient sample. Patients were only recruited in the Oregon Hearing Research Center, which is a tertial referral center. Patients stem predominantly from the Pacific Northwest of the United States. Thus, the documented sample in the database cannot be considered representative of the world-wide patient population. Third, the database does not include any validated measurements for tinnitus severity, which have been published in the meantime, e.g., the tinnitus handicap inventory (THI) [22]. The THI is a widely used questionnaire validated to assess tinnitus-related, self-reported handicaps with high test-retest reliability [23] and has been cross-validated with other questionnaires like the tinnitus questionnaire (TQ) [24]. In contrast, when the Oregon database was developed, only relatively rough measurements of tinnitus severity were available. Fourth, the last update of the database was in 1994. Thus, advances in tinnitus research in the last decade with a stronger focus on finding a cure for tinnitus result in additional requirements for a modern database, which are only insufficiently met by the existing *Oregon Tinnitus Data Archive*. A new international database, including patients from many centers in many countries, containing cross-sectional as well as longitudinal data from intervention studies would largely facilitate future tinnitus research. An essential requirement for the establishment of such an international database would be a standardized method of data collection and documentation, as well as standardized treatment intervention. Up to now, there has been great variability in each of the centers in the assessment of tinnitus patients. For the assessment of tinnitus severity, many different tinnitus questionnaires are in use and psycho-acoustical measurements are performed with various techniques and methods jeopardizing the comparability and pooled analyses of data from different centers. Since there is a great need for the further development of new treatment strategies, improving already available treatment options and allowing individualised treatment approaches based on reliable predictors, the establishment of an international tinnitus database has been initiated in the framework of the Tinnitus Research Initiative (TRI). The primary aims of this database are:

• Subtyping of different forms of tinnitus, based on their specific symptoms and/or their response to treatment modalities (e.g. by cluster analysis [25])

• Identifying predictors for treatment response to specific treatments

• Assessment of treatment outcome for specific treatments using a modular approach

• Identification of candidate clinical characteristics for delineating neurobiologically distinct forms of tinnitus

• Explanation of discrepant results of different studies (e.g. by the possibility to identify differences between the study populations)

• Collection of epidemiological data

• Cross-validation of different assessment instruments in different languages

• Development of an individualized treatment algorithm for every single patient based on the individual diagnostic profile

• Delineation of subgroups with similar characteristics and generating data about discriminative power of diagnostic procedures

## Materials and methods

### Standardized data collection

In order to assure comparability of the data collected in different centers, a common standard must be defined which all participating centers have to follow. The standard for the TRI database is based on a consensus for patient assessment and outcome measurement found by tinnitus experts from many countries during an international tinnitus conference in Regensburg in 2006 [26]. The participants at this conference agreed that for the sake of inter-study comparability, a minimum of standardized assessments during diagnostic and therapeutic evaluation of tinnitus is needed. Methods for tinnitus patient assessment and treatment outcome measurement in the database were chosen according to this consensus. These core assessments consist of a detailed tinnitus and medical history, otological examination, psycho-acoustic measures of tinnitus and a variety of validated questionnaires assessing tinnitus severity and quality of life and became part of standardized case report forms (CRF). The CRF is now available in a number of languages such as English, Flemish, French, German, Italian, Portuguese, Spanish. In most cases validated translations of the questionnaires were available and included in the CRF. If necessary, translations were provided. Following the lead of accepted translation procedures [27,28] translators had to be experts in the medical field of tinnitus, native speakers of the target language (e.g. Italian) and fluent in the source language (i.e. English). These experts provided a forward translation that was checked by a second person with the same level of expertise. In case of occasional disagreements, these were resolved by consensus (reconciliation process). The TRI database allows for such an analysis of the psychometric properties of these newly translated questionnaires.

Data from all forms of treatment interventions (e.g., pharmacological, psychotherapeutic, auditory or brain stimulation interventions, etc.) will be entered into the database. Treatment forms included so far in the database encompass pharmaceutical interventions (63% of all patients), brain stimulation techniques (i.e., transcranial magnetic or electrical stimulation; 23% of all patients) and cognitive behavioral therapeutic approaches (about 12%). In the future, further treatments may be added to the database. The only preconditions are that treatment interventions have to be clearly defined and performed in a standardized way. According to various durations of the different treatment interventions, the CRF was organized flexibly in terms of both the number of visits and the intervals between visits. Also the use of additional assessment methods is supported by the database. However, the core assessments are identical for all visits and all interventions to allow cross-comparison. Table 1 gives an example of a standardized CRF in English and how it is used for a pharmacological trial of 12 weeks' duration. The design of the CRF allows the documentation of both cross-sectional and longitudinal data. Up to now, 13 centers from 8 countries (Argentina, Brazil, Belgium, France, Germany, Italy, Spain, and New Zealand) have participated in the database project. CRFs are currently available in English, Flemish, French, German, Italian, Portuguese and Spanish. Translations into further languages are being prepared. Finally, the database is open to anyone who is interested in participating in the project (either by collecting data or addressing research questions and accessing the whole dataset). Scientific agreements define the rights and duties of each participating center. As a general rule, every participating center has full access to its own dataset. In addition, each center may have access to the whole dataset under predefined conditions. Research questions and access to the whole dataset will be discussed within the TRI database scientific committee and has to be approved by it. Further information on how to participate may be found on http://database.tinnitusresearch.org or by sending an email to database@tinnitusresearch.org.

**Table 1:** Overview of the standardized content of a CRF used for pharmacological studies according to the consensus. Measurements at each visit are graded according to essential to collect (A) or highly recommended (B).

	Screening	Baseline	Week 2	Week 4	Week 8	Week 12 = End	Week 16 Follow up
**TSCHQ**	A						

**Otological Examination**	A						

**Medical history**	A						

**Audiometry**	A	A				B	

**Loudness match**		B				B	

**Pitch match**		B				B	

**Maskability**		B				B	

**Residual Inhibition**		B				B	

**THI**	A	A	A	A	A	A	A

**TBF 12**	B	B	B	B	B	B	B

**Tinnitus Numeric Rating Scales**	A	A	A	A	A	A	A

**BDI**	B	B	B	B	B	B	B

**WHOQOL**	B	B	B	B	B	B	B

**CGI (change)**			A	A	A	A	A

**Concomittant medication**	A	A	A	A	A	A	A

**Adverse Events**			A	A	A	A	A

**Comorbidity**	A	A	A	A	A	A	A

### Database construction and technical details

A novel approach to entering data for anonymous patients has been created with strict observation of the guidelines of Good Clinical Practice (GCP) and Federal Drug Administration (FDA) regulations. This approach defines each patient with a unique hash code made up of a 40 cipher string. Since this string is not really legible in routine use for data entry, a substitute rule has been defined to identify the patient more easily: a combination of the patient number (6 ciphers) and the center identification number (ID) (3 ciphers), linked by a hyphen. Thus, in all cases the patient relative to the database was anonymous, and the only people able to recognize the patients behind the numbers were the data entry staff members.

The environment surrounding this approach had been made of a v5.x MySQL database and a PHP v4.x and - after migration onto a more modern hosting server - v5.x based application using some JavaScript functionality (scarcely). For all database entries, modification, and updating modes, PHP-based transactions have been newly designed so as to enable both a complete usage and error tracking system. Thus, revisions and other changes were easy to follow and made up one part of the users' administration in order to modify the users' activity (e. g. active, inactive, banned).

So as to minimize the efforts to manually enter all CRFs, a system was designed using the German application FormPro^® ^to automatically scan the CRFs through a high-volume scanner and import the recognized data (after some corrections, if necessary) into the database.

In order to optimize and simplify data evaluation, some of the most relevant calculations had been put into the system first hand. The rest of the data structures were adapted to fit the mathematical needs of data analysis.

In order to not be jeopardized by any kind of data loss or unwanted data change, the system was finalized by a separately developed backup system, that both incrementally and completely did its job every night and put the one-day evaluation backup file (packed and encrypted) on a separate, specially secured FTP (SFTP) server for downloading by the data evaluation staff.

The final enrichment of the system was done by the development of a criteria-based validation system: Only validated data was incorporated into the evaluation backup file.

Although initially the use of a virtual private network (VPN) had been recommended, a https://approach using encrypted user logins and passwords was considered safe. For security reasons, however, the hardening of the underlying Linux operating system (openSuSE 10.3, later 11.1) was undertaken so as to prevent any kind of external hacking or cracking trials. Furthermore, the system's logging function was set from debug (9) to paranoid mode (10) automatically sending the log files to the system administrator for both automatic and manual tracking.

### Data handling/quality

High emphasis has been placed on the standardization of data collection and on assuring the quality of data handling. After completing a manual CRF data entry, each center sends the original CRF to the central database management at the University of Regensburg, where the digital data entry is conducted. A copy of each CRF remains in the individual study center. The Center for Clinical Studies at the University Hospital in Regensburg developed a data validation strategy and generated a detailed data handling plan, which defines the action to be taken in case of missing values, implausible or illegible data, incomplete data or self-evident corrections according to GCP guidelines. The data handling plan is the foundation for data entry and contains several approaches to ensure the validity of the data entered.

In addition, automatic computer-based checks during data entry (i.e. defined value ranges, field type controls) and regular manual checks of missing and implausible values in the database minimize errors and upgrade the data quality. Based on subject identification numbers, each missing and implausible value can be located and a query to the relevant study center is generated (query-management).

### Statistical analysis approaches

The primary goal of the database is the definition of subgroups of tinnitus patients, who respond to a specific treatment according to their tinnitus-specific characteristics and concomitant medical conditions. Analyses start with descriptive statistics using counts, proportions (percentages), means and standard deviations, medians and ranges. The focus will be on a backward-oriented analysis strategy aiming to characterize patients responding to any intervention. Responders will be determined via changes in their tinnitus scores from baseline to end of treatment, mostly after 8 or 12 weeks. The criteria of minimally important clinical changes will be established by cross-validation of the different assessment instruments.

Responders will be compared to non-responders in order to detect differences in demographic or clinical variables. These analyses will be performed for responders versus non-responders across therapies as well as for each single intervention separately. Significant factors indicating treatment response can then be tested in the future in specifically designed studies. Due to the exploratory nature of the project and the large amount of potential important variables, the a priori specification of a detailed statistical analysis plan is not feasible. The statistical analysis plan will be developed as the data base grows and results from initial analyses become available. Ideally, results will be the basis for a decision support tool using a set of pre-defined clinical and demographic criteria that will help to tailor the proper therapy for a given patient.

## Discussion

The TRI database is unique in that it is the first database containing standardized collected data from patients undergoing different types of treatment interventions. Study centers from many different countries contribute to the database. Data entered in the database ranges from highly selected patient populations participating in controlled trials to unselected patients populations receiving standard treatments under real world conditions. Finally, participation in the database project is open to anyone who is willing to follow the guidelines of data collection (i.e., using the standardized CRF). Thus, this database enables the comparability of study results from different clinical trials, from different centers as well as from patient populations with differing ethnic backgrounds for the first time. The prerequisite of such an international research project is a standardized method of data collection. This standard repertoire of tinnitus assessment methods is based on a consensus conference [26], and has been agreed upon by all participating centers. Still these assessment tools always represent a trade-off between comparability of measurements from different centers and the most sensitive measurements, which may not be available in all centers and which depend on the intervention being studied. Nevertheless, the search for predictors for different treatment responses is very much dependent on the availability of large patient samples, which can only be achieved in a reasonable time by pooling data from different centers. Also the generalization of results requires representative samples but also reliable and not too complicated assessment procedures. Therefore, such a compromise is necessary. Furthermore, the present set of measurements implemented in the database represents a core set that can be used in all centers, but the database has been constructed in a most flexible modular structure, so that additional instruments such as results from functional imaging may be included at any time in the future. On the other hand, if it turns out that some measures have become outdated for e.g., scientific reasons, these may be removed from the database, if this is suggested by the TRI database scientific committee.

Another major problem of tinnitus research is that tinnitus is a subjective experience (like pain), which is not accessible by the use of objective measurement methods. It can only be quantified by using self-rating questionnaires. This new database contains the most widely used tinnitus questionnaires and therefore offers the possibility to perform advanced analyses on the psychometric properties (reliability, validity, sensitivity) of these measures. One comparison for such evaluation attempts may be the Clinical Global Impression Scale (CGI; [29]), which is also used in this database and represents a validated research tool for assessing efficacy of treatment studies. Also, new questionnaires, which have been specifically developed to assess change in tinnitus severity, may be incorporated into the database as soon as they are available and can be cross-validated with other questionnaires in a large patient sample.

Taken together, the TRI database represents a new, unique research tool and can potentially facilitate the clinical characterisation of tinnitus subtypes, the detection of predictors of individual responses to treatment and may help individualise and improve treatment approaches in the future.

## Abbreviations

TSCHQ: Tinnitus Sample Case History Questionnaire; THI: Tinnitus Handicap Inventory; TBF 12: Tinnitus Impairment Questionnaire; BDI: Beck Depression Inventory; CGI: Clinical Global Impression Scale; WHOQOL: World Health Organization - Quality of Life (Questionnaire)

## Competing interests

The authors declare that they have no competing interests. The ManaThea GmbH is a private company specialized on the development of medical software solutions and provides the technical support within this project (development of the software, maintenance and service of the server hosting the database, etc.). The ManaThea GmbH has no competing interests.

## Authors' contributions

All authors contributed to the writing of the manuscript. ML, BL, GH developed, designed and coordinated the international research network of tinnitus experts, which provide clinical data sets for the database. MM programmed the software, data handling and entry is managed by YE, SS, JR. Statistical analyses are performed by MK and FZ. All authors have read and approved the final manuscript.

## Pre-publication history

The pre-publication history for this paper can be accessed here:

http://www.biomedcentral.com/1472-6947/10/42/prepub
